# Accelerated failure-time model with weighted least-squares estimation: application on survival of HIV positives

**DOI:** 10.1186/s13690-021-00617-0

**Published:** 2021-05-31

**Authors:** Yesuf Abdela Mustefa, Ding-Geng Chen

**Affiliations:** 1 Computing and Analytics Directorate, Technology and Innovation Institute of Ethiopia, Addis Ababa, Ethiopia; 2 Department of Statistics, University of Pretoria, Pretoria, South Africa

**Keywords:** Survival data analysis, Accelerated failure-time, Cox proportional-hazards regression, Weighted least-squares estimation, Heteroscedasticity

## Abstract

**Background:**

*Survival analysis is the most appropriate method of analysis for time-to-event data. The classical accelerated failure-time model is a more powerful and interpretable model than the Cox proportional hazards model, provided that model imposed distribution and homoscedasticity assumptions satisfied. However, most of the real data are heteroscedastic which violates the fundamental assumption and consequently, the statistical inference could be erroneous in accelerated failure-time modeling. The weighted least-squares estimation for the accelerated failure-time model is an efficient semi-parametric approach for time-to-event data without the homoscedasticity assumption, which is developed recently and not often utilized for real data analysis. Thus, this study was conducted to ascertain the better performance of the weighted least-squares estimation method over the classical methods.*

**Methods:**

*We analyzed a REAL dataset on Antiretroviral Therapy patients we recently collected. We compared the results from classical methods of estimation for the accelerated failure-time model with the results revealed from the weighted least-squares estimation.*

**Results:**

*We found that the data are heteroscedastic and indicated that the weighted least-square method should be used to analyze this data. The weighted least-squares estimation revealed more accurate, and efficient estimates of covariates effect since its confidence intervals were shorter and it identified more significant covariates. Accordingly, the survival of HIV positives was found to be significantly linked with age, weight, functional status, CD4 (Cluster of Differentiation agent 4 glycoproteins), and clinical stages.*

**Conclusions:**

*The weighted least-squares estimation performed the best in providing more significant effects and precise estimates than the classical accelerated failure-time methods of estimation if data are heteroscedastic. Thus, we recommend future researchers should utilize weighted least-squares estimation rather than the classical methods when the homoscedasticity assumption is violated.*

**Supplementary Information:**

The online version contains supplementary material available at 10.1186/s13690-021-00617-0.

## Background

Although the Cox proportional-hazards (PH) model [1] is the most employed technique in survival analysis because of its reduced set of assumptions about the baseline hazard function, formulation of the accelerated failure-time (AFT) model [2] allows the derivation of a time ratio, which is more interpretable than a ratio of two hazards [3]. The AFT model doesn’t require PH assumption which is seldom met assumption of the Cox PH model [3]. It also encompasses relatively a wide range of survival time distributions and yields more powerful estimates than the Cox PH model, provided that method imposed assumptions satisfied [4]. Thus, the AFT model is more appealing in many ways [5].

Conventionally, the rank [6–13] and least-squares [14–17] are most often used methods of inference for the AFT model [18]. The classical AFT models in general are unified by the adoption of a log-linear representation with a particular survival time distribution for the error term [4]. These classical methods impose constant variance (homoscedasticity) assumption and hence, don’t take heteroscedastic data into consideration. Consequently, rank or least-squares estimation (LSE) based inference for heteroscedastic data is not reliable [5, 18]. The coverage probabilities of the 95% confidence intervals (CI) of the estimated coefficients are considerably lower than the nominal level 0.95 because the variance estimates of the parameter estimators are mostly under-estimated with the homogeneous assumption of variance. Besides, it results in the loss of efficiency for coefficient estimators. Moreover, the intercept estimation is inconsistent.

To incapacitate these negative aspects, Yu, et al. [18] proposed the weighted least-squares estimation (WLSE) for the AFT model. It is a semi-parametric approach to handle both homoscedastic and heteroscedastic data beyond the incorporation of censoring, which was shown to be more efficient theoretically as well as extensive Monte-Carlo simulation studies. Specifically, Yu, et al. [18] showed that the WLSE was more efficient than the classical AFT method with higher statistical power to detect the associated statistical significance with lower standard errors of estimates and narrower confidence intervals due to lower variances obtained from synthetic observations combined with the actual observations.

Therefore, this article is aimed to promote the utilization of this WLSE model in analyzing our HIV data to detect significant effects of covariates on HIV patients’ survival for a more valid inference and conclusions. In doing so, we compared the classical AFT method with WLSE to ascertain the validity, detective ability, and efficiency of inference from WLSE based on real Antiretroviral Therapy (ART) dataset with more covariates. The magnitude of standard errors of estimates, the width of confidence intervals, and the number of significant covariates was considered as comparison criteria due to the WLSE has been shown to be efficient and can recover the true effect of the parameters and their variances.

## Data and research method

### Description of the dataset

The data was obtained from University of Gondar referral hospital ART database. Patients whose ART starting date unrecorded were excluded. On the other hand, a total of 3042 patients with complete records of their baseline characteristics were targeted for the study. We used systematic random sampling since a complete list was available. To determine the sample size, we used Cochran’s [19] formula $n=\frac{Z_{0.025}^2\pi \left(1-\pi \right)/{E}^2}{1+\frac{Z_{0.025}^2\pi \left(1-\pi \right)/{E}^2}{N}}$ where:- *n* is the number of patients required for the study; ${Z}_{0.025}^2$ is the upper 25^*th*^ percentile point of the standard normal distribution and it is 1.96; *π* is the proportion of death in the study population which was 0.17; *E* = 0.05 was margin of error; and *N* was the total number of patients with complete records of baseline characteristics. Accordingly, a sample of 203 HIV patients who started ART between 2003 and 2009 were followed until April 2015.

The response variable was considered to be the length of time measured in months from ART initiation until time of death (censor).

#### Covariates of the study

There were six covariates recorded at the beginning of ART, namely “gender” with two levels (male and female); “functional status” with three levels (working, ambulatory, and bedridden); WHO “Clinical Stage” with four levels (I, II, III, and IV); Cluster of Differentiation Agent 4 (CD4) percent with two levels (12–15%, and 16–28%), age and weight. We used CD4 percent instead of CD4 count because it was recorded in the database. It was recorded in the form of class intervals for some of the patients and in the form of actual values for the others. Thus, we used the Struges [20] rule to determine the appropriate number of class groupings. However, we merged all percent categories greater than or equal to 16% since the percentages of death in those categories were very small. Similarly, we merged the first three categories of the WHO clinical stage.

### Statistical methods

#### The log-rank test

We used the log-rank test to determine which covariates to select for further analysis. The log-rank test, developed by Mantel and Haenszel [21], is a non-parametric test for equality of survival functions in two or more groups. Let *t*_*i*_ be times where events are observed (assume these are ordered and there are *D* such times); *d*_*ik*_ be the number of observed events from group *k* at time *t*_*i*_; *Y*_*ik*_ be the number of subjects in group *k* that are at risk at time *t*_*i*_, ${d}_i=\sum \limits_{j=1}^n{d}_{ij}$*,* ${Y}_i=\sum \limits_{j=1}^n{Y}_{ij}$, and *n* is the number of comparison groups. Then to test the hypothesis, a vector *Z* is computed, where the *k*^*th*^ element is ${Z}_k=\sum \limits_{i=1}^D\left({d}_{ik}-{Y}_{ik}\frac{d_i}{Y_i}\right)$. The test statistic $Q={Z}^{\mid }{\hat{\Sigma}}^{-1}Z$ where $\hat{\Sigma}$ is variance covariance matrix obtained from the data follows chi-square distribution with *n* degrees of freedom. Larger value of the test statistic or the corresponding smaller *P*-value provides strong evidence against the null hypothesis of no difference.

#### Assessment plots

We used plots to assess the time-varying nature of covariates; heteroscedasticity in the data; and the linearity assumption of covariates. Plots of coefficients of covariates over time help examine the time-varying nature of covariates [4]. Reference lines at zero falling within the 95% confidence limits provide evidence that covariates are not time-varying. Plot of the estimated variances versus the means can be used to assess heteroscedasticity in the data [18]. Any pattern suggests the presence of heteroscedasticity. Plot of the log-survival time against a covariate can be used to assess the linearity assumption and a straight Locally Weighted Scatterplot Smoothing (LOWESS) line close to a horizontal reference line shows no violation to the linearity assumption [4].

#### The classical AFT model

The AFT model regresses survival time $\mathcal{T}$ on covariates X as follows,

$$ {T}_i\equiv \log \left({\mathcal{T}}_i\right)={\alpha}_0+{\beta}_0^T{X}_i+{\in}_i,\kern0.5em i=1,2,\dots, n $$ Eq. 1.

Where *α*_0_ is the true intercept; *β*_0_ is the true p-dimensional vector of slope parameters; the index “*i*” corresponds to the study participant in the analysis and *n* is the total number of patients. Let $\tilde{X}=\left[1\kern0.5em X\right]$ and ${\tilde{\beta}}_0^T=\left[{\alpha}_0\kern0.5em {\beta}_0^T\right]$ where ${\tilde{\beta}}_0$ is the vector of coefficients for the AFT model [18].

In this model, let ∈_*i*_ = *σe*_*i*_. Then, *e*_*i*_ is the error term which is independently and identically distributed (IID) with unspecified distribution function F of mean 0 and variance 1. In other words, ∈_*i*_ is an IID error term with a constant variance *σ*^2^ (homoscedasticity) assumption and a particular survival time distribution [5]. The most commonly used survival distributions for AFT metrics are exponential (Exp), Weibull (Weib), log-logistic (Logl), lognormal (LN), and generalized gamma (GG) [3]. The exponential distribution is the special case of the Weibull distribution. Similarly, the generalized gamma distribution includes a wide range of family distribution as its special case. Its flexible hazard function allows for many possible shapes such as Weibull, exponential, and lognormal distributions with various values of the shape and the scale parameters [4]. Therefore, we used the GG AFT model for evaluating and selecting an appropriate model from its parametrically nested possible AFT models for the dataset by testing the shape and the scale parameters.

Moreover, we used information criterion statistics (ICS), −2 log *likelihood* (−2LL) for comparison of the performance of parametrically nested classical AFT models and Akaike information criterion (AIC) for comparison the performance of other alternative classical AFT models. Accordingly, the best model was the one with the smallest ICS indicating the minimum loss of information.

Finally, the performance of the best-fitted classical AFT model was compared with the model fitted by the WLSE. This comparison was based on either the magnitude of standard errors (SE) of estimates or the length of confidence intervals to determine the accuracy and efficiency of a given inference. The ability to detect more significant covariates was another interest of comparison.

#### The weighted least-squares method

The WLSE method frees the homoscedasticity assumption in Eq. 1, which is the most practical method for real data analysis.

Define *Z* = min {*T*, *C*} and *δ* = *I*(*T* ≤ *C*), where C is the logarithm of the censoring time, T is as defined in Eq. 1, *I*(⋅) is the indicator function. Then, the triplet denoted by {*Z*_*i*_, *X*_*i*_, *δ*_*i*_} represents values of a transformation of observed survival time (*Z*_*i*_), covariates (*X*_*i*_), and censoring indicator function (*δ*_*i*_) for the *i*^*th*^ patient where *C*_*i*_ is assumed to be independent of *T*_*i*_ and *X*_*i*_. The synthetic observation is defined to be ${T}_i^{\ast }={Z}_i{\delta}_i+E\left({T}_i|{T}_i>{C}_i\right)\left(1-{\delta}_i\right)\kern0.5em i=1,2,\dots, \mathrm{n}$, where, *E*(.) is the expectation function. The WLSE utilizes a weighted least-squares equation as in Yu, et al. [18] with synthetic observations weighted by the square root of their variances where the variances are estimated via the local polynomial regression. Thus, the weighted regression according to Yu, et al. [18] is as follows.

$$ {T}_{inew}^{\ast }={\alpha}_0{x}_{i0 new}+{\beta}_0^T{X}_{inew}+{e}_i,\kern0.5em i=1,2,\dots, n $$ Eq. 2.

Where ${T}_{inew}^{\ast }={T_i}^{\ast }/{\sigma}_{NPE}\left({\mu}_i\right)$; *σ*_*NPE*_(*μ*_*i*_) is the non-parametric estimator (NPE) via the local polynomial regression of *σ*^∗^(*μ*_*i*_), the square root of the variance of ${T}_i^{\ast }$; *x*_*i*0*new*_ = 1/*σ*_*NPE*_(*μ*_*i*_); *X*_*inew*_ = *X*_*i*_/*σ*_*NPE*_(*μ*_*i*_); *e*_*i*_,'*i*’, and n are as defined in Eq. 1.

Based on the weighted least-squares regression in Eq. 2, all ${\tilde{\beta}}_0$, including *α*_0_, are slope parameters.

## Results

Covariates with a probability of being significantly less than or equal to 10% in the log-rank test were potentially considered for further analysis besides age and weight. The *P*-value for gender is greater than 0.1(10% probability of inclusion) (Table 1). Thus, it was not further included in the models. Since age and weight were numeric, we tested them one at time (independently) using all survival models considered in this study and we found them significant at 5% level of significance for further analysis. The averages of age and weight for the study patients were 31 years old and 45 Kg respectively.

**Table 1:** Patients Characteristics

Covariates	Labels	Number of Patients	Number of Deaths	LRT
				P-Value
Gender	Female	118	21	1.000
	Male	85	14	1.000
FS	Working	136	15	0.002
	Ambulatory	55	14	0.002
	Bedridden	12	6	0.002
CS	I, II, & III	154	13	0.000
	IV	49	22	0.000
CD4%	12–15%	162	33	0.100
	16–28%	41	2	0.100

*FS: Functional Status; CS: Clinical Stage; CD4%: Cluster of Differentiation Agent Four in Percent; LRT: Log-Rank Test.*

**Fig. 1:**
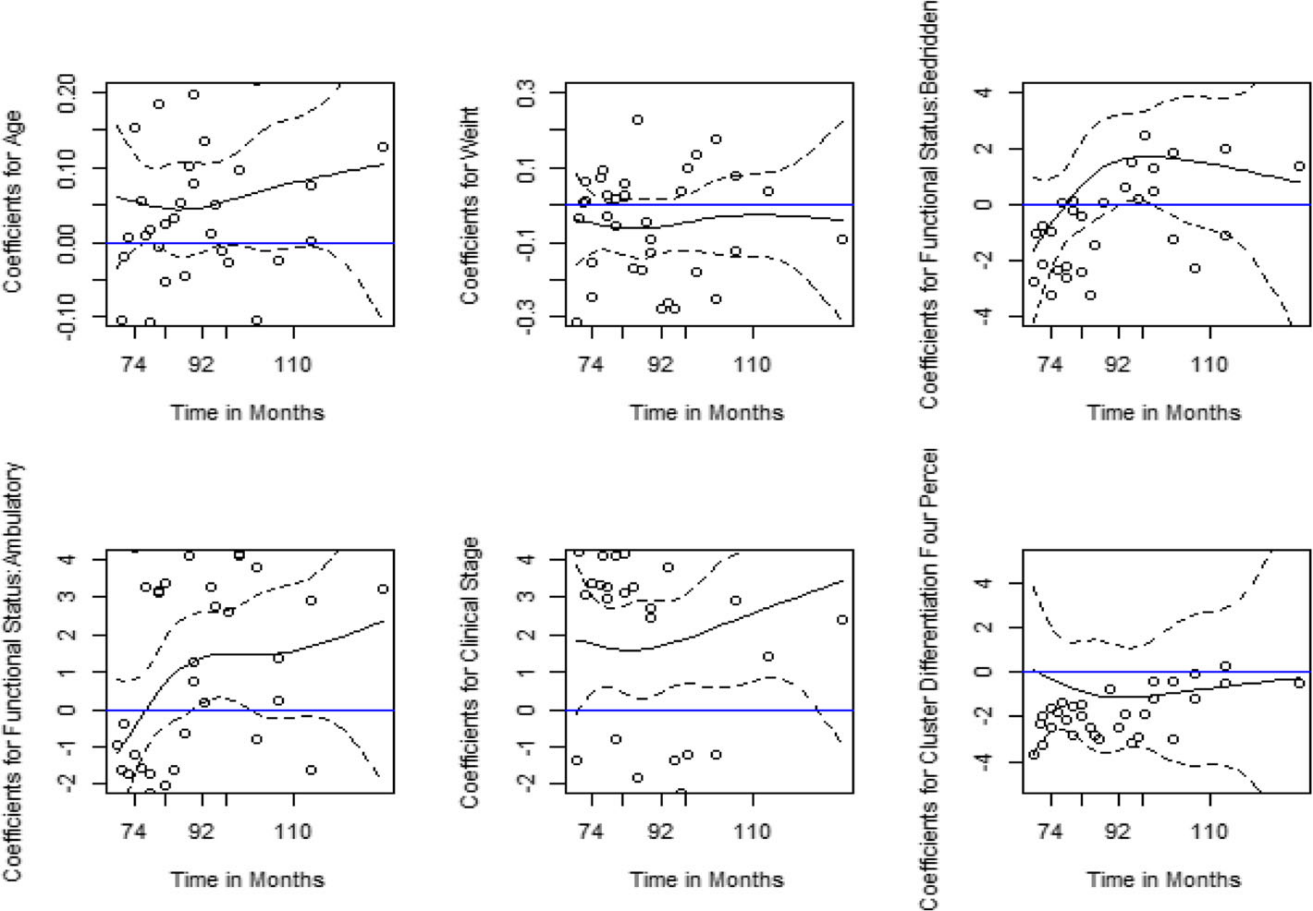
Plots of Coefficients over Time

We proceeded further with an assessment of the time-varying nature of covariates in Fig. 1. We used the Cox PH model for this test. We found that the reference line on zero differences falls within the 95% confidence intervals for age, weight, and CD4%, which confirms that these covariates are not time-varying.

**Fig. 2:**
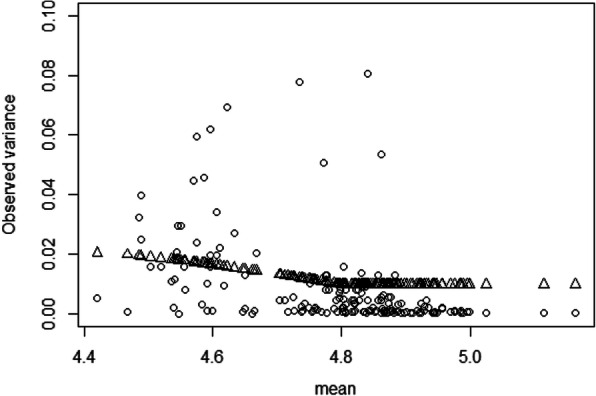
Variance function estimated from the weighted least-squares method. The dots are the observed variances and the triangles are the estimated variances

We also examined the plot of the estimated variances versus the means from the weight least-squares method, as shown in Fig. 2. The data was found to be heteroscedastic, which violates the assumption of the classical AFT models.

**Fig. 3:**
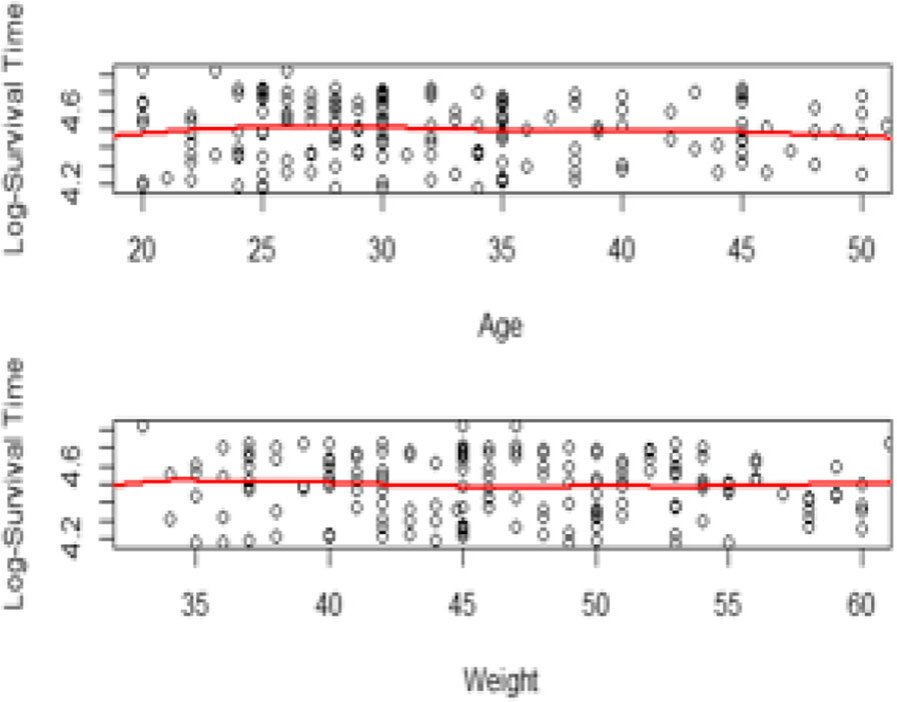
Linearity Assumptions for Age and Weight

We further examined the linearity assumption using locally weighted scatter plot smoothing (LOWESS). As seen in Fig. 3, there were no violations to the covariates “age” and “weight”. Thus, we used the values of age and weight as they were rather than their transformed form. Furthermore, there was no significant interaction effect to be included in the models.

According to Yu, et al .[18], the WLSE is a valid inference in such cases. We summarized the results in Table 2. The reference categories are working for functional status; I, II, & III for clinical-stage; and 12–15% for CD4%. The level of significance is considered to be 5%.

**Table 2:** Weighted Least-squares Estimation

Covariates	Estimates	SE	95% CI	
			LCL	UCL
Constant	4.773	0.026020	4.722	4.824
Age	−0.00516	0.000010	−0.00518	− 0.00514
Weight	0.00497	0.000005	0.00495	0.00498
BFS	−0.04742	0.013663	−0.07420	−0.02064
AFS	−0.02048	0.003439	−0.02721	−0.01374
CS (IV)	−0.25080	0.002214	−0.25513	−0.24646
CD4%(16–28)	0.10320	0.046816	0.01143	0.19496

*Estimates: Coefficients Correspond to the log Median Survival Time; Estimate for Age corresponds to a year increase in Age; Estimate for Weight corresponds to a Kilogram increase in Weight; CI: Confidence Interval; LCL: Lower Confidence Limit; UCL: Upper Confidence Limit; BFS: Bedridden Functional Status and its reference category is Working Functional Status; AFS: Ambulatory Functional Status and its reference category is Working Functional Status; CS (IV): Clinical Stage IV and its reference category is Clinical Stage I-III; CD4%16–28: Cluster of Differentiation Agent Four Percent 16–28 and its reference category is Cluster of Differentiation Agent Four Percent 12–15.*

In addition to the WLSE, we revealed the results from the classical methods namely, parametric AFT model, rank, and least-squares estimation (LSE) to ascertain the superiority of WLSE over the classical methods. We selected the appropriate parametric AFT model for the dataset among the exponential, Weibull, log-logistic, lognormal, and generalized gamma AFT models.

The shape parameter (Q) from the classical GG AFT model in Table 3, is not significantly different from 0 or 1. This indicates that the LN and the Weibull AFT models are likely to be appropriate among the special cases of GG AFT model. However, Q is estimated to be 0.411 and it is nearer to 0 than to 1. For this conclusion, we selected the LN AFT model over the Weibull. Moreover, we considered ICS in Table 4 for selecting the most appropriate model.

**Table 3:** Estimates of Shape Parameter (Q) from the Generalized Gamma AFT Model

Estimates	95%CI	
	LL	UL
0.411	−0.507	1.328

*CI: Confidence Interval; LL: Lower Limit; UL: Upper Limit.*

**Table 4:** Information Criterion Statistics for Classical AFT Models

ICS	Exp	Weib	Logl	LN	GG
-2LL	479.7	380.6	379.9	379.8	379.2
AIC	485.7	390.6	389.9	389.8	391.2

*ICS: Information Criteria Statistics; Exp: Exponential; Weib: Weibull; Logl: Log-logistic; LN: Log-Normal; GG: Generalized Gamma; LL: Log-likelihood; AIC: Akaike Information Criterion.*

The smaller magnitude of the information criterion statistics (ICS) for LN AFT model led us to the conclusion that the LN AFT model is the best fit for the data. Therefore, we used the LN AFT model to represent the parametric AFT models for comparison. Furthermore, the estimation performance of the other parametric AFT models was the same as LN AFT model as in Table 5.

**Table 5:** Estimates from the Classical AFT and the Cox PH Models

Method	Covariates	Estimates	SE	95%CI	
				LCL	UCL
LN AFT	meanlog	4.832	0.1564	4.526	5.139
	sdlog	0.232	0.0286	0.182	0.295
	Age	−0.009	0.0029	− 0.015	−0.003
	Weight	0.008	0.0032	0.002	0.014
	CS (IV)	−0.278	0.0599	−0.395	− 0.160
Rank	Age	−0.009	0.0033	−0.015	−0.002
	Weight	0.008	0.0033	0.001	0.014
	CS (IV)	−0.277	0.0572	−0.389	− 0.165
LSE	Age	−0.009	0.0027	−0.014	−0.004
	Weight	0.008	0.0026	0.003	0.013
	CS (IV)	−0.272	0.0530	−0.376	− 0.168
Cox PH	Age	0.054	0.0177	0.019	0.089
	Weight	−0.047	0.0228	−0.092	− 0.002
	CS (IV)	1.917	0.3684	1.195	2.639

*Estimates: Coefficients Correspond to the log Median Survival Time for the First Three and log Hazard for the Cox PH; Estimate for Age corresponds to a year increase in Age; Estimate for Weight corresponds to a Kilogram increase in Weight; SE: Standard Error; CI: Confidence Interval; LCL: Lower Confidence Limit; UCL: Upper Confidence Limit; LN AFT: Log-Normal Accelerated Failure-Time; CS: Clinical Stage; LSE: Least Square Estimation; PH: Proportional-Hazards; meanlog: Mean of Log Survival Time; sdlog: Standard Deviation of Log Survival Time.*

As seen in Table 5, the performance of the three classical methods was similar in this particular study. Accordingly, age, weight, and WHO clinical stage were significant based on these methods. However, an efficient method is obviously more likely to identify significant covariates. Thus, we found the LSE to be the most efficient among the classical methods in Table 5 since the standard errors (SE) associated with its estimates are relatively small. Similarly, LN AFT model was more efficient as compared to the rank method. The Cox PH model identified the same significant covariates as the classical AFT models. However, directions of the effects differed in the Cox PH model since estimates were affected by the time-varying nature of clinical stage.

Nevertheless, we compared the performance of LSE with the WLSE since the LSE was the best among the classical methods. The results in Table 2 revealed that the WLSE is more accurate than the LSE. It resulted in efficient estimates of covariates effect on HIV patients’ survival since the narrow CI indicates a relatively small standard error of estimates as theoretically showed in Yu, et al. [18]. It also identified more significant covariates since functional status and CD4 percent were additional significant covariates that were not identified by LSE. Though we considered the same set of covariates in all models at the beginning, we included only significant covariates in the final models as per their ability to identify significant effects. We interpreted the results of WLSE as follows.

Holding the effects of all other factors constant, the log survival time for a patient with an additional year of age decreases by 0.00516. In other words, the median survival time of a patient is *e*^0.00516^ = 1.01 times as compared to that of a patient with a single year older. This indicates that the survival probability of younger patients is better than older. Studies by [22–24] confirmed that the hazard of death increases with higher age intervals. These studies also confirmed that less weight, low level of CD4, higher clinical stage, and non-working functional status are associated with increased hazard rate. Similarly, we found that the logarithm of survival is 0.00497 more for a kilogram of additional weight. For patients with bedridden and ambulatory functional status, it is respectively 0.047 and 0.0205 less as compared to those with working functional status. Moreover, it is 0.251 less for patients at clinical stage IV than patients at lower stages and it is 0.1032 more for patients with CD4 percent of 16–28% as compared to those with lower CD4 percent. In our study, gender of patients was not significantly associated with their survival at 5% level significant. In contrast, it was found to be significant according to the three studies we mentioned in this discussion. Note that, interpretations in terms of median time ratio can be obtained by exponentiation of the corresponding coefficient estimates as it is indicated for age.

## Discussions

With many available statistical software packages, such as ‘survival’ and ‘flexsurv’ in R, STATA, SAS, Python, etc., AFT models have become popular in survival data analysis with a wide range of clinical and epidemiological applications. Among others, AFT models have been utilized in acute liver, cancer, and HIV AIDS studies [3, 22–25]. However, the validity of an inference depends on the realization of model assumptions. One of the assumptions in AFT models is constant variance assumption. According to Yu, et al .[18], a valid inference can be obtained from the WLSE than the classical AFT methods when the constant variance assumption is violated. Moreover, the WLSE for the AFT model is constructed operationally by the synthetic observations based on the transformation that allows the same conditional expectations as the logarithm of survival time [26] so that the transformation has no effect on the manner of interpretation for AFT model. Therefore, the degree of precision obtained from WLSE is superior to the classical methods without any difficulties imposed on the interpretation of effects. However, we relied on evidence from previous studies about the efficiency of the WLSE and utilized only magnitudes of standard errors, the width of confidence intervals, and the number of identified significant covariates as criteria of comparison. Thus, future researchers can consider more criteria than we used for comparison of the efficiency of WLSE with that of classical AFT methods.

## Conclusions and recommendations

We utilized AFT models based on the classical and WLSE methods on a real ART dataset we recently compiled with more covariates than considered in [18]. Among possible parametric AFT models, the lognormal AFT model fitted the data well. We compared the results from this model with the results from LSE and rank methods. From the classical methods, LSE was found to be the best; LN AFT the second; and rank the least. Consequently, we compared LSE with WLSE since WLSE was proved in Yu et al. [18] to be an efficient estimation. The width of confidence intervals from the WLSE was found to be shorter than that of the classical methods. The WLSE also detected more significant covariates. As a result, the WLSE performed best in providing more significant effects and precise estimates. However, the data was heteroscedastic. Thus, we recommend future researchers extend the application of WLSE to a homoscedastic real dataset with more covariates to ascertain its validity. They should utilize WLSE rather than the classical AFT methods when the homoscedasticity assumption is violated to obtain efficient estimates. Moreover, health workers should be more cautious when a patient is in advanced clinical stages, old in age, relatively lower in weight, in bedridden or ambulatory functional status, or with lower CD4 percent during ART initiation.

## Supplementary Information

**Additional file 1.**

## Data Availability

All data analyzed during this study are included in this published article as supplementary material with the file name “13690_2021_617_MOESM1_ESM.csv”.

## Abbreviations

AFS: Ambulatory Functional Status
AFT: Accelerated Failure-Time
AIC: Akaike Information Criterion
ART: Antiretroviral Therapy
BFS: Bedridden Functional Status
CD4: Cluster of Differentiation Agent 4
CI: Confidence Intervals
CS: Clinical Stage
EXP: Exponential
FS: Functional Status
GG: Generalized Gamma
ICS: Information Criterion Statistics
IID: Independently and Identically Distributed
Kg: Kilogram
LCL: Lower Confidence Limit
LL: Log-Likelihood
LN: Log-Normal
Logl: Log-logistic
LOWESS: Locally Weighted Scatterplot Smoothing
LSE: Least-Squares Estimation
NPE: Non-Parametric Estimator
PH: Proportional Hazards
SE: Standard Errors
UCL: Upper Confidence Limit
Weib: Weibull
WHO: World Health Organization
WLSE: Weighted Least-Squares Estimation
